# Prognostic value of γ‐aminobutyric acidergic synapse-associated signature for lower-grade gliomas

**DOI:** 10.3389/fimmu.2022.983569

**Published:** 2022-11-03

**Authors:** Hongxiang Jiang, Zhiqiang Sun, Fei Li, Qianxue Chen

**Affiliations:** ^1^ Department of Neurosurgery, Renmin Hospital of Wuhan University, Wuhan, Hubei, China; ^2^ Central Laboratory, Renmin Hospital of Wuhan University, Wuhan, Hubei, China

**Keywords:** γ-aminobutyric acidergic synapses, tumor immune microenvironment, risk signature, prognostic factors, lower-grade glioma

## Abstract

**Background:**

Synapse-associated proteins (SAPs) play important roles in central nervous system (CNS) tumors. Recent studies have reported that γ-aminobutyric acidergic (GABAergic) synapses also play critical roles in the development of gliomas. However, biomarkers of GABAergic synapses in low-grade gliomas (LGGs) have not yet been reported.

**Methods:**

mRNA data from normal brain tissue and gliomas were obtained from the Genotype-Tissue Expression (GTEx) and The Cancer Genome Atlas (TCGA) databases, respectively. A validation dataset was also obtained from the Chinese Glioma Genome Atlas (CGGA) database. The expression patterns of GABAergic synapse-related genes (GSRGs) were evaluated with difference analysis in LGGs. Then, a GABAergic synapse-related risk signature (GSRS) was constructed with least absolute shrinkage and selection operator (LASSO) Cox regression analysis. According to the expression value and coefficients of identified GSRGs, the risk scores of all LGG samples were calculated. Univariate and multivariate Cox regression analyses were conducted to evaluate related risk scores for prognostic ability. Correlations between characteristics of the tumor microenvironment (TME) and risk scores were explored with single-sample gene set enrichment analysis (ssGSEA) and immunity profiles in LGGs. The GSRS-related pathways were investigated by gene set variation analysis (GSVA). Real-time PCR and the Human Protein Atlas (HPA) database were applied to explore related expression of hub genes selected in the GSRS.

**Results:**

Compared with normal brain samples, 25 genes of 31 GSRGs were differentially expressed in LGG samples. A constructed five-gene GSRS was related to clinicopathological features and prognosis of LGGs by the LASSO algorithm. It was shown that the risk score level was positively related to the infiltrating level of native CD4 T cells and activated dendritic cells. GSVA identified several cancer-related pathways associated with the GSRS, such as P53 pathways and the JAK-STAT signaling pathway. Additionally, CA2, PTEN, OXTR, and SLC6A1 (hub genes identified in the GSRS) were regarded as the potential predictors in LGGs.

**Conclusion:**

A new five-gene GSRS was identified and verified by bioinformatics methods. The GSRS provides a new perspective in LGG that may contribute to more accurate prediction of prognosis of LGGs.

## Introduction

Gliomas are heterogeneous and invasive tumors of the central nervous system (CNS) that are derived from glial cells. Gliomas have poor prognosis (1). In 2016, gliomas were categorized as classes I–IV by the World Health Organization (WHO), and the use of the biomarkers isocitrate dehydrogenase (IDH) mutations and 1p/19q co-deletion was introduced (2). Among gliomas, diffuse low-grade (grade II) and intermediate-grade (grade III) gliomas constitute low-grade gliomas (LGGs). There are significant differences in clinical behavior and prognosis among LGGs (3). LGG patients usually experience seizures, but gliomas can also lead to neurological and neurocognitive impairment and even premature death in the course of the disease. Among LGGs, IDH has similar prognostic utility (4). The molecular subtype of the tumor determines the outcome of LGG surgical resection, which is positively correlated with the degree of tumor malignancy (5).

In the past decade, our comprehension of the molecular pathogenesis of gliomas has improved greatly. Unfortunately, however, this comprehension has not translated into better treatments for patients, which highlights the still existing gaps in our knowledge. More research studies of LGG and its biomarkers are needed to develop better treatments.

The microenvironment of gliomas contains non-neoplastic cells such as neurons, glial cells, immune cells, and vascular cells. All these cells can promote and support the growth of tumors (6). The CNS is also rich in neurotransmitters, which create a unique microenvironment for brain tumors, where neurotransmitter-mediated intracellular signaling pathways can be transduced by cancer cells and induce cancer cell growth, activation, and metastasis (7). With the progressive discovery of glioma synapses and metastatic neuronal synapses, it is believed that neurotransmitters may play crucial roles in tumor growth, and the speculation that tumor cells may stimulate their innervation has been confirmed (8, 9). It has been suggested that microenvironment interaction, especially the abnormal interaction between glial cells and synapses, is one of the neuropathological mechanisms underlying Rett syndrome, Down syndrome, spinal muscular atrophy, and other diseases (10). More and more recent studies have found that the communication between neurons and glial cells is related to several neuropsychiatric and neurodegenerative diseases, such as schizophrenia (11).

In high-grade gliomas (glioblastoma and grade III astrocytoma), glioma cells are depolarized by excitation signals from neuronal glioma synapses (NGSs), and these signals are amplified through gap junctions to promote their proliferation (12). In addition, targeting neuroligin-3, a key synaptogenic factor, significantly reduced the growth of gliomas (13). Although the role of synapse-associated proteins (SAPs) in breast metastasis and the development of high-grade gliomas has been recognized, their role in the development of LGGs is still unclear. Hence, a more systematic study of SAPs from more angles is needed to better understand their roles in LGGs.

For malignant glioma tissue with neuronal interaction, glioma cell culture is conducive to the tumor microenvironment (TME) to enhance self-proliferation and escape from immune response (14). Relevant reports have demonstrated that gliomas could control normal neuronal plasticity and developmental factors in the TME, so the abnormal connections between neurons and tumor cells could be established through glioma synapses (15). At the same time, through neuronal glioma synapse (NGS)-mediated depolarization of the calcium signaling network in glioma cells, the electrical activity of neurons can increase tumor proliferation and invasion and lead to the progression of glioma (6). Previous reports have shown that glutamatergic synapses are considered to be related to the progression of intracerebral gliomas (13).

When exploring the abnormal glutamatergic synapses in the glioma microenvironment, similar attention has also been paid to the dysregulated γ-aminobutyric acidergic (GABAergic) signaling and its role in the progression of brain tumor-associated glioma and epilepsy (16). Recently, it has been reported that B cells can release γ-aminobutyric acid (GABA), a well-known neurotransmitter molecule, which could promote the differentiation of monocytes into anti-inflammatory macrophages, thus secreting interleukin-10 (IL-10), and thereby inhibiting the anti-tumor CD8 T-cell response. The GABA secreted by B cells may become a new direction of tumor immunotherapy (17, 18). Nevertheless, GABAergic signal transduction related to gliomas has not been reported. The existing literature shows that the GABA A receptor expressed by glioma cells is functional, and that endogenous GABA A receptor activity inhibited the proliferation of glioma cells (19). In addition, after adult glioblastoma stem cells lost their tumorigenicity, the production and secretion of 4-hydroxybutyric acid (a by-product of GABA catabolism) increased, resulting in decreased cell invasiveness (20).

It can be inferred that the interaction between GABAergic synapses and the immune state has a special role in the prognosis of gliomas. To confirm this hypothesis, we developed and verified a new GABAergic synapse-related risk signature (GSRS), which may promote the understanding of glioma progression and provide a novel idea for the study of biomarkers for effective diagnosis and prognosis. Additionally, we also studied the correlation between risk signals and immune characteristics in LGG.

## Materials and methods

### Samples from public data

Genome-wide RNA-seq expression data as well as clinical and molecular information were collected from the TCGA database^1^ and used as a training dataset. WHO grade II–III gliomas were included. Any cases that had inadequate clinical or missing prognostic information were excluded.

LGG samples were selected from parts A and B of the CGGA database^2^. They were then integrated, standardized, and utilized as a validation dataset. Samples from patients with a 30-day survival rate or no survival data were excluded from this study because these patients were more likely to die from other life-threatening conditions (e.g., stroke and heart failure) than from the LGG.

We selected 453 LGG specimens from TCGA (training dataset) and 590 LGG specimens from CGGA (validation dataset) for further analysis. The mRNA expression data of all these LGG specimens were complete and clinical data were attached. A control collection of 1,137 normal brain samples (containing tissues from various areas of the brain such as cortex, brainstem, and cerebellum) with full mRNA-seq data was also employed. We utilized the “normalize Between Arrays” function of the R software package “limma” to reduce various batch effects when combining the mRNA-seq data of TCGA and genotype-tissue expression (GTEx), as well as CGGA parts A and B (21, 22).

### Clinical tissue samples

All patients whose tissues were utilized gave their informed consent. Between March 2020 and April 2022, we obtained 5 control samples from patients with intracerebral hemorrhage and an additional 12 LGG samples. Before surgery, none of the gliomas had been treated with chemoradiotherapy. Related mRNA expression of GSRS hub genes in LGG was verified using independent samples from our institution. This protocol was authorized by the Ethics Committee of the Renmin Hospital of Wuhan University (Wuhan, Hubei, China).

### Obtaining GABAergic synapse-related gene sets

A total of 31 GABAergic synapse-related gene (GSRG) sets, “GOBP_NEGATIVE_REGULATION_OF_SYNAPTIC_TRANSMISSION_GABAERGIC” and “GOBP_POSI TIVE_REGULATION_OF_SYNAPTIC_TRANSMISSION_GABAERGIC,” were obtained from the Molecular Signatures Database^3^.

### Differentially expressed genes between normal tissues and LGGs

The GTEx and TCGA-LGG databases were applied for the training dataset. The R software package “limma” (22) was used for discovery of differentially expressed genes (DEGs) from GSRGs. The criteria were a false discovery rate < 0.05 and an absolute value of log fold change (logFC) > 1.

### Protein–protein interaction network analysis

A protein–protein interaction (PPI) network with 31 GSRGs was created using the STRING database^4^. Nodes with interaction connection confidences > 0.4 are shown.

### Genomic alterations of 31 GSRGs

Copy number variation (CNV) deep deletion, CNV amplification, missense mutations, truncating mutations, in-frame mutations, and fusions of 31 GSRGs were explored using the cBioPortal dataset^5^.

### Construction of GSRS

The “survival” R software program was used to analyze the predictive value of GSRGs in LGG (*p*-value < 0.05 was the threshold for further investigation). Survival status, survival time, and expression levels of prognosis-related genes in LGG patients were calculated by the least absolute shrinkage and selection operator (LASSO) regression algorithm (23) (penalty parameter λ was selected based on 10-fold cross-validation). Then, the gene and its regression coefficient were determined based on the most suitable λ value.

The risk score was calculated according to the following formula:

Risk score = exprgene (1) × coefficientgene (1) + exprgene (2) × coefficientgene (2) + · · · + exprgene(*n*) × coefficientgene(*n*)

where *n* is the number of prognostic genes in the risk signature, coefficientgene is the coefficient of the gene, and exprgene is the expression value of the gene.

### Principal components analysis

All LGG samples were categorized into low- and high-risk groups by the estimated median risk score. Principal components analysis (PCA) was performed on dimensionality reduction of mRNA expression data in TCGA-LGG to confirm between-group differences and CGGA.

### Predictive role of GSRS

For both training and validation datasets, we assessed the prognostic relevance of the GSRS in LGG by Cox regression analysis and Kaplan–Meier survival curves. Furthermore, we computed not only the area under the receiver operating characteristic curve (AUC-ROC) for GSRS, but also other clinical risk variables for predicting the overall survival (OS) in LGG patients. Three categories of AUC-ROC for measuring the accuracy of a diagnostic technique were defined as follows: poor accuracy (0.5 < AUC-ROC ≤ 0.7), moderate accuracy (0.7 < AUC-ROC ≤ 0.9), and high accuracy (0.9 < AUC-ROC ≤ 1) (24).

### Clinicopathological characteristics of GSRS

Patients enrolled in the study were classified into high- and low-risk categories in the training and validation cohorts. The chi-square test was used for difference analysis of the risk score for clinicopathological parameters such as age, gender, histology, WHO glioma grade, IDH mutational status, and 1p19q co-deletion status. A *p*-value < 0.05 was deemed significant.

### Profiles of tumor-infiltrating immune cells

The CIBERSORT algorithm was applied to determine the abundance profile of immune cells in the low- and high-risk groups individually. The link between the proportion of tumor-infiltrating immune cells (TIICs) and risk score in the training and validation datasets was explored using Pearson correlation analysis and the Wilcoxon test. The stromal score, immune score, and tumor purity of each LGG sample were also determined using the “estimate” package, based on the ESTIMATE method (25).

### Single-sample gene set enrichment analysis

The critical genes of 29 immune-related pathways were obtained from the related literature (26). According to melanoma mRNA transcripts per million (TPM) data, the single-sample gene set enrichment analysis (ssGSEA) was applied to assess the level of TIICs (27). In addition, a differential analysis of gene hallmark enrichment degree with 29 types of immune-related hallmarks was done between low- and high-risk groups. We also explored the expression levels of immune checkpoints (ICPs) and immunogenic cell death (ICD) modulators in low- and high-risk groups, given their role in cancer immunity.

### Mutational status analysis

Somatic tumor mutational burden (TMB) was computed in the TCGA-LGG dataset as the total number of mutations found in each sample. Additionally, the R software package “maftools” was utilized to investigate the predictive usefulness of TMB in LGG, comparing low- and high-risk groups and calculating mutational status.

### Gene set variation analysis

The Molecular Signatures Database was used to download and choose sets of marker genes that summarize and reflect unique, well-defined biological states or processes with consistent expression. The R software package “GSVA” was used to implement gene set variation analysis (GSVA) of signature gene sets in TCGA-LGG for low- and high-risk groups (28).

### Verification of hub genes of GSRS

Kaplan–Meier survival curves were applied to identify the prognosis-related genes of the GSRS. Furthermore, the Human Protein Atlas (HPA) database^6^ was used to examine the protein levels of genes identified in normal brain and LGG tissue.

### RNA extraction and quantitative real-time PCR

RNA extraction of prognosis-related genes from tissues and cells was performed using TRIzol reagent (Invitrogen, Carlsbad, CA, United States). The RNA was then tested for purity as well as concentration. A reverse transcription kit was applied in the conversion of whole samples to cDNA. Performing qRT-PCR by employing the SYBR Green system. GraphPad 7 was used to assess statistics. The results from the experimental and control groups were compared using the relative Ct technique, with GAPDH serving as an internal reference.

## Results

### Genetic differences of GSRGs in LGGs

**Figure 1:**
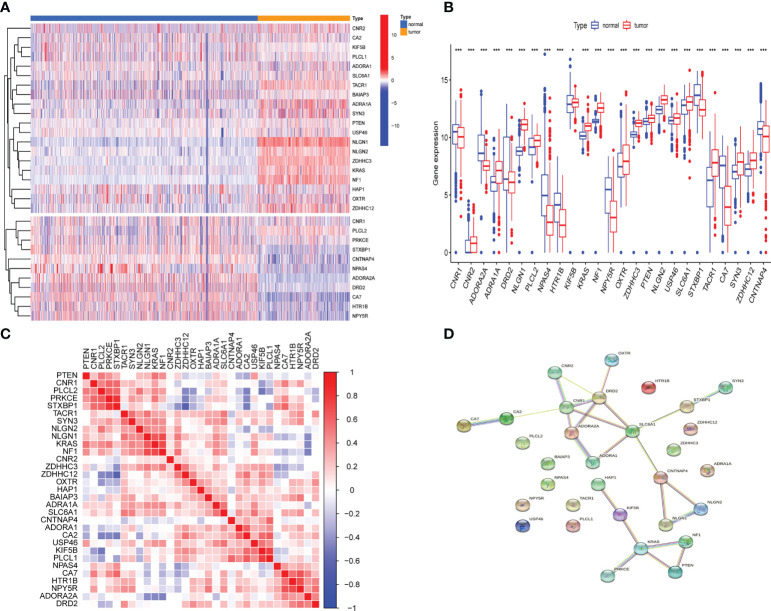
The genomic characterization of GABAergic synapse-related genes (GSRGs). **(A)** Heatmap for differentially expressed GSRGs; genes with red color are involved in positive regulation of GABAergic synaptic transmission, while genes with blue color mainly participate in negative regulation of GABAergic synaptic transmission. **(B)** Boxplot for differentially expressed GSRGs. **(C)** Correlation plot for GSRGs; red squares indicate positive correlation and blue squares indicate inverse correlation. **(D)** Protein–protein interaction network of GSRGs in the STRING database. ***p < 0.001, *p < 0.05.

Differential analysis of 31 GSRGs (**Figure 1A**) showed that 25 genes (**Figure 1B**) were differentially expressed between LGG and normal samples in the training dataset. **Supplementary Table 1** includes downregulated and upregulated GSRGs and the related logFC values. Additionally, a strong correlation in expression among GSRGs was found by Spearman’s correlation analysis (**Figure 1C**). Furthermore, the co-expression correlation among GSRGs was confirmed by PPI network analysis (**Figure 1D**). Subsequently, to further explore the genomic identities of GSRGs in LGG, using mutational analysis the cBioPortal database was applied to reveal the copy number polymorphisms and somatic mutational status of GSRGs (**Supplementary Figure 1**).

### Verification of GSRS

**Figure 2:**
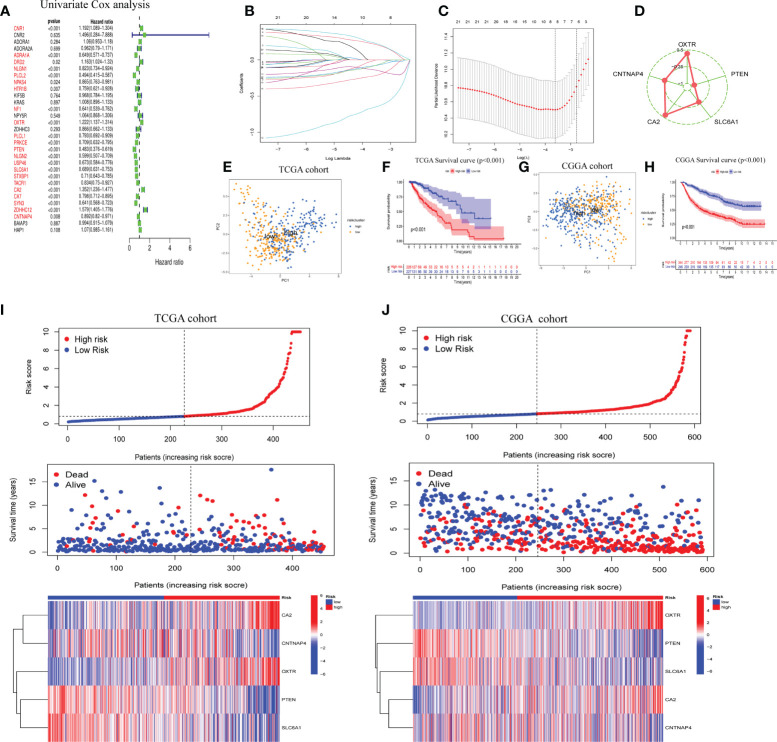
Construction of five-gene GABAergic synapse-related risk signature (GSRS). **(A)** Forest plot for the survival analysis of LGG patients using a univariate Cox model after adjustment for GSRGs; red color represents *p* < 0.05. **(B)** The craft plot for partial likelihood deviance in LASSO; different colors represent different genes in GSRS. **(C)** Partial likelihood deviance as a function of regularization parameter λ in the training dataset. Each red point marks a λ value along regularization paths, and gray error bars represent confidence intervals for the cross-validated error rate. The left vertical dotted line marks the minimum error, whereas the right vertical dotted line marks the most significant λ value, the error of which is within 1 SD of the minimum. The horizontal row of numbers above the plot marks the gene number in each condition upon shrinkage and selection based on linear regression. **(D)** Radar diagram of efficiency of the five genes in GSRS; the closer the red dot is to the outside, the greater the value it represents. **(E)** Principal components analysis (PCA) of LGG samples in TCGA; dots in blue represent samples in high-risk groups and dots in yellow represent samples in low-risk groups. **(F)** Overall survival analysis of risk score for LGG patients in TCGA. **(G)** PCA in CGGA-LGG. **(H)** Survival analysis in CGGA-LGG. According to training **(I)** and validation **(J)** sets, the distribution of risk score, corresponding OS, and gene expression are listed in the picture from top to bottom.

LASSO regression analysis was performed after first identifying five prognosis-related genes from the 31 GSRGs using univariate Cox analysis (**Figure 2A**). After validation with LASSO analysis, the best-fitting model featured the following five genes: OXTR, PTEN, SLC6A1, CA2, and CNTNAP4. These five genes and their corresponding coefficients are summarized in **Supplementary Table 2**. According to the mRNA expression level of each risk gene and these corresponding coefficients, the risk score for each patient was calculated (**Figures 2B** **–D**).

As determined by PCA, the median of the GSRS sufficiently distinguished low-risk and high-risk clusters. Additionally, the prognoses between the low- and high-risk groups were also shown distinctly by survival analysis performed on the training and validation datasets (**Figures 2E** **–H**). Furthermore, the risk score, survival time, and risk gene expression were plotted for the GSRS of the TCGA-LGG and CGGA cohorts (**Figures 2I, J**). Taken together, these data showed that GSRS-based risk scores may be better predictors of prognosis in LGG patients compared to other clinical factors.

### Predicting prognosis of LGGs with new risk scores

**Figure 3:**
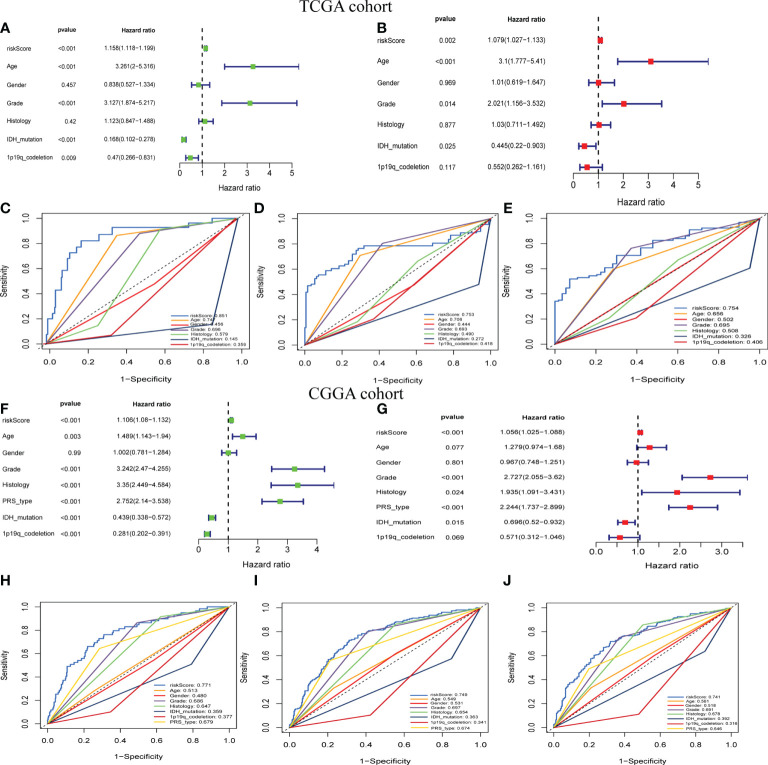
The prognostic value of GSRS. **(A, B)** In the training set, the forest plot on the left is for the univariate and multivariate Cox analysis evaluating the association of the risk score and clinical factors with patient OS. The ROC curve of risk score and clinical factors for predicting 1-year **(C)**, 3-year **(D)**, and 5-year **(E)** OS. **(F, G)** In the validation set, univariate and multivariate Cox analysis of risk score and clinical factors. ROC curve of risk score compared with other clinical factors for predicting 1**-**year **(H)**, 3-year **(I)**, and 5-year **(J)** OS.

To further examine the potential role of GSRS in independently predicting prognosis, univariate and multivariate Cox analyses were performed (**Figures 3A, B**). We found that the risk score could be an independent predictor of OS for the TCGA-LGG cohort (*p* < 0.001). The risk score had a greater AUC-ROC compared to all clinical factors related to prognosis such as age, gender, histology, WHO glioma grade, IDH mutational status, or 1p19q co-deletion status. The AUCs for risk score for 1, 3, and 5 years in the training dataset were 0.885, 0.753, and 0.754, respectively (**Figures 3C** **–E**). The same conclusion was also validated by the CGGA-LGG cohort, and the hazard ratio (HR) of the risk score from multivariate Cox regression analysis was 1.056 (**Figures 3F, G**, *p* < 0.001). The AUCs for risk score for 1-, 3-, and 5-year OS in CGGA-LGG were 0.771, 0.749, and 0.741, respectively (**Figures 3H** **–J**).

### Correlations between clinicopathological features and GSRS

**Figure 4:**
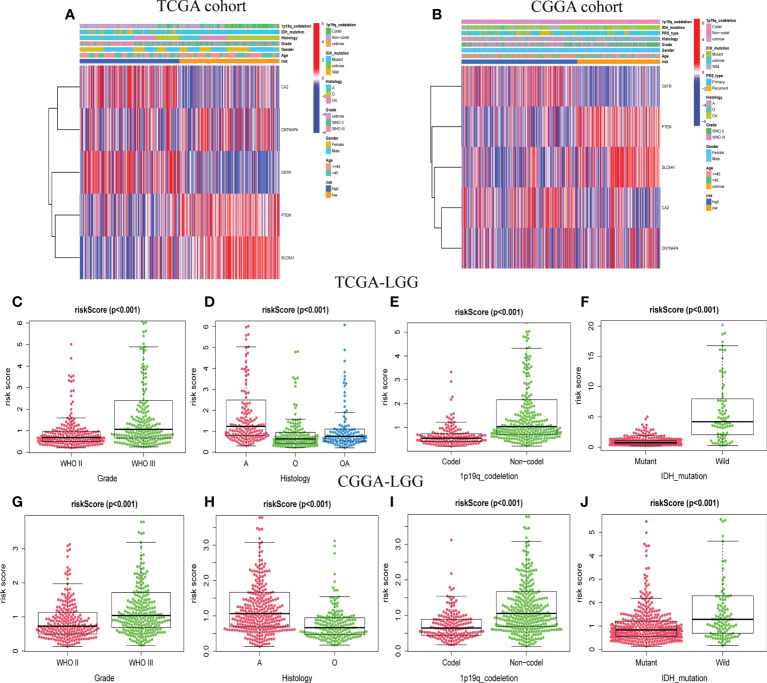
The association between risk score and clinicopathological factors. Heatmap of the correlations between risk score and clinicopathological characteristics of LGG in TCGA **(A)** and CGGA **(B)** cohorts. Distribution of GABAergic synapses-related risk signature among LGG patients stratified by WHO grade, histology, IDH status, 1p/19q co-deletion status, and gender in TCGA **(C–F)** and CGGA **(G–J)** cohorts.

For the training and validation datasets, respectively, 453 and 590 cases with valid data for age, gender, WHO glioma grade, IDH mutational status, and 1p19q co-deletion status were screened out. To explore the distribution of clinical factors among the different risk groups, chi-square tests were conducted using the R function “chisq.test”. **Table 1** shows the differences between the TCGA and CGGA cohorts. GSRS-based risk scores were obtained and were significantly correlated with WHO glioma grade, IDH mutational status, and 1p19q co-deletion status in the datasets (**Figures 4A, B**). In particular, LGG samples with higher WHO grade, IDH wild type, or 1p19q non-co-deletion showed higher risk scores than the other samples; differences were also seen among the LGG and other samples for histology (**Figures 4C** **–J**). Therefore, the risk score values were associated with the histology, WHO grade, IDH mutational status, and 1p19q co-deletion status of LGG.

**Table 1:** Correlation between five GSRS genes’ risk scores and clinicopathological factors of glioma patients in the two cohorts.

Features	Training set TCGA RNA-seq cohort (*n* = 453)			Validation set CGGA RNA-seq cohort (*n* = 590)		
	Low-risk score(*n* = 227)	High-risk score(*n* = 226)	*p*	Low-risk (*n* = 246)	High-risk score(*n* = 344)	*p*
**Age**			0.002			0.780
≤45	144	127		172	260	
>45	83	99		74	84	
**Gende**r			0.913			0.025
Female	100	101		113	137	
Male	127	125		133	207	
**Grade**			<0.001			<0.001
II	144	79		143	126	
III	83	147		103	218	
**IDH status**			<0.001			<0.001
Wild type	10	71		36	102	
Mutant	217	155		210	242	
**1p/19q Co-deletion**			<0.001			<0.001
Yes	125	31		124	96	
No	102	195		122	248	

### Profiles of tumor-infiltrating immune cells

**Figure 5:**
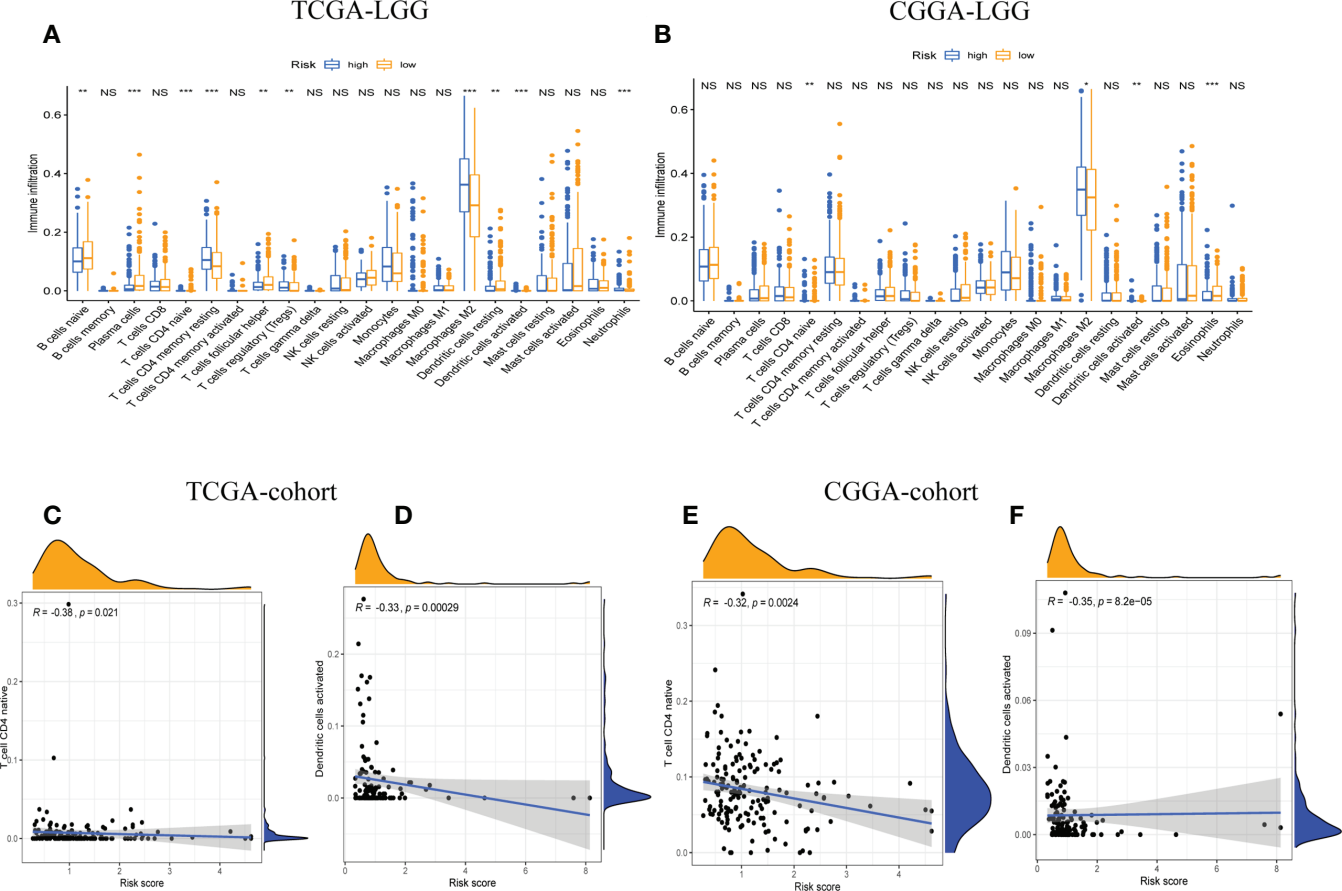
The correlation between tumor-infiltrating immune cells (TIICs) and GSRS. Difference analysis of 22 kinds of abundance of TIICs, immune score, and stromal score in low- and high-risk groups in training **(A)** and validation sets **(B)**. Spearman’s correlation analysis between risk score and M0 macrophages and CD4 memory resting T cells in TCGA **(C, D)** and CGGA cohorts **(E, F)**; each dot plot represents a subject, and the correlation is fitted into a straight blue line. R, rho; NS, Non Significance, ***p < 0.001, **p < 0.01, *p < 0.05.

The relative proportions of 22 types of immune cells were examined, based on calculations from the “CIBERSORT” algorithm. The results of differences between the low-risk group and high-risk group were presented as boxplots (**Figures 5A, B**). The low-risk group had significantly higher infiltration of native CD4 T cells and activated dendritic cells than the high-risk group. In addition, the stromal score recorded the presence of stromal cells in the tumor tissue, and the immune score indicated the infiltration of immune cells into the tumor area. The samples from the high-risk group showed higher glioma-associated immune and stromal scores than those from the low-risk group. High-risk LGG samples showed higher infiltration levels of stromal and immune cells. Furthermore, we confirmed that native CD4 T cells and activated dendritic cells were significantly negatively associated with risk scores, as shown by correlation analysis (**Figures 5C–F**). It suggested that the risk scores may be correlated with the prognosis of LGG patients in terms of decreased native CD4 T cells and activated dendritic cells.

**Figure 6:**
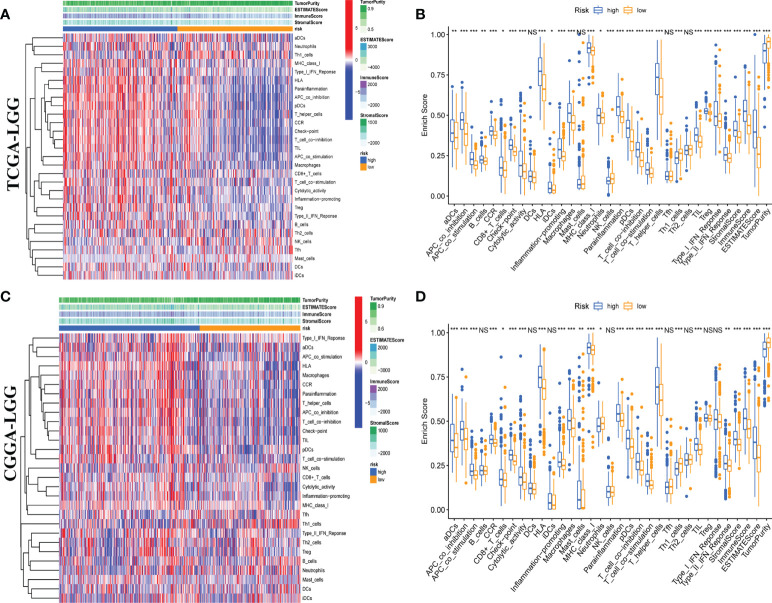
Single-sample gene set enrichment analysis (ssGSEA) of immune hallmarks. Heatmap of ssGSEA scores among low- and high-risk groups in training **(A)** and validation **(C)** sets. Boxplot of ssGSEA scores, stromal score, immune score, and tumor purity among low- and high-risk groups in TCGA **(B)** and CGGA **(D)** cohorts. NS, Non Significance, ***p < 0.001, **p < 0.01, *p < 0.05.

Moreover, we quantitatively assessed the activities and abundances of pathways, functions, or immunocytes according to the ssGSEA scores. As expected, more infiltrating immune cells and activity of immune-related pathways were seen in samples with higher ssGSEA scores. As shown in the heatmaps (**Figures 6A, C**) and boxplots (**Figures 6B, D**), high-risk samples were correlated with higher ssGSEA scores for most immune cell types. In general, high-risk LGG patients are more likely to have a higher fraction of TIICs and more active immune-related pathways than the others. In the high-risk group, the TIICs (native CD4 T cells and activated dendritic cells) were much lower than the baseline levels.

### Correlation between immune modulators and risk score

**Figure 7:**
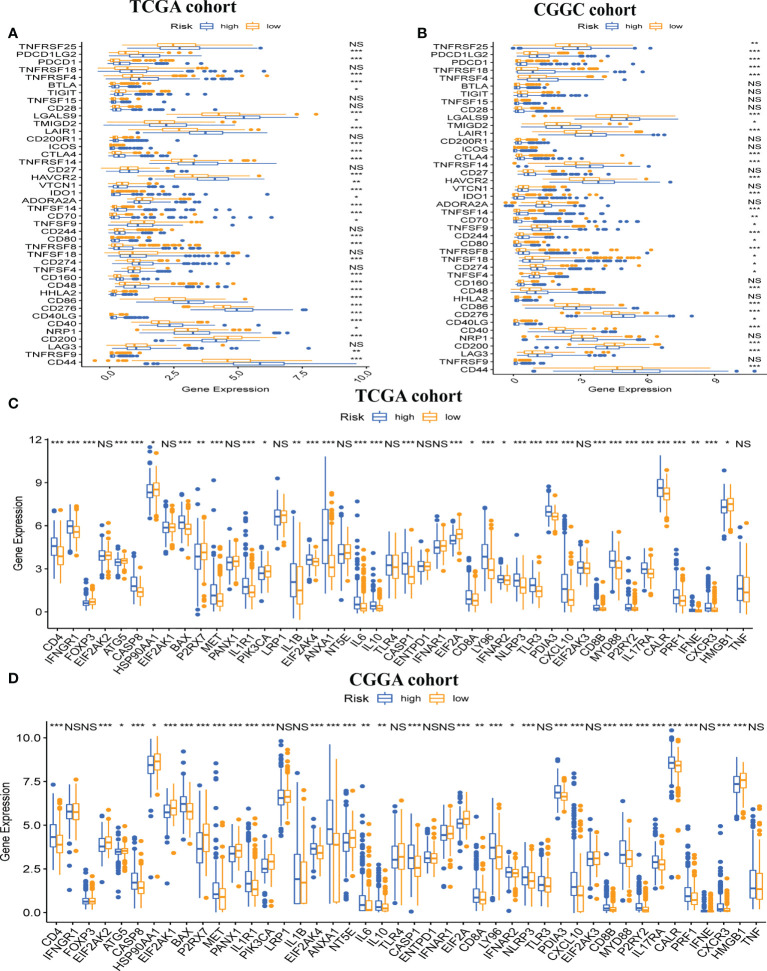
Association between risk subtypes and ICPs and ICD modulators. Differential expression of ICP genes among the risk subtypes in **(A)** TCGA and **(B)** CGGA cohorts. Differential expression of ICD modulator genes among the risk subtypes in **(C)** TCGA and **(D)** CGGA cohorts. NS, Non Significance, ***p < 0.001, **p < 0.01, *p < 0.05.

Next, we explored gene expression levels in different risk populations, taking into account the significance of ICP and ICD regulators in anticancer immunity. There were 46 ICP-related genes identified in the training and validation datasets, and 42 of these genes were found in the TCGA and CGGA cohorts (**Figures 7A, B**), with different expressions in different risk groups. Critically, key ICPs such as CTLA4, PDCD1 (programmed death receptor-1 [PD-1]), and CD274 (PD-L1) were highly upregulated in the high-risk group. Similarly, there were 34 DEGs for ICD in the TCGA group and 33 DEGs for ICD in the CGGA group (**Figures 7C, D**). Hence, the risk score not only can show the expression level of ICPs and ICD modulators, but also can serve as a potential immunotherapy biomarker.

### Correlation between risk score and mutational status

**Figure 8:**
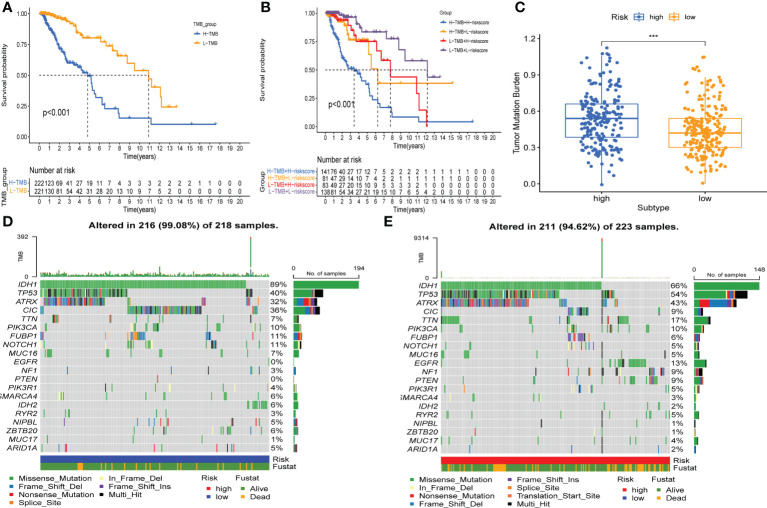
Association between risk subtypes and TMB and mutation. **(A)** Survival analysis of TMB and OS of the patients with LGG in TCGA. **(B)** K–M curves of TMB combined with risk score in TCGA-LGG. **(C)** Difference analysis of TMB among low- and high-risk subtypes in LGG patients. **(D)** Top 20 highly mutated genes in the LGG low-risk group. **(E)** Top 20 highly mutated genes in the LGG high-risk group. ***p < 0.0001.

Firstly, we determined the prognostic value of TMB in LGG. For the survival analysis in TCGA-LGG, the patients who had higher TMB were more likely to have a worse prognosis than those with lower TMB (**Figure 8A**). At the same time, the Kaplan–Meier curve of risk score combined with TMB indicated that a higher risk score and higher TMB had the worst OS, while those with lower risk score and lower TMB had the best prognosis (**Figure 8B**). Hence, we found an association between risk score and TMB in LGG. The difference analysis of TMB between low- and high-risk groups showed a significant positive association for TMB and risk score (**Figure 8C**). Subsequently, mutations were shown in low - and high-risk populations. The mutation frequencies of IDH1, TP53, and ATRX were the highest in 20 genes studied in each subtype (**Figures 8D, E**). These findings indicate that GSRS-based risk scores could predict the TMB and somatic mutation rates in LGGs, and the higher risk score group may have a positive anticancer immune response.

### Conducting GSVA between different groups

**Figure 9:**
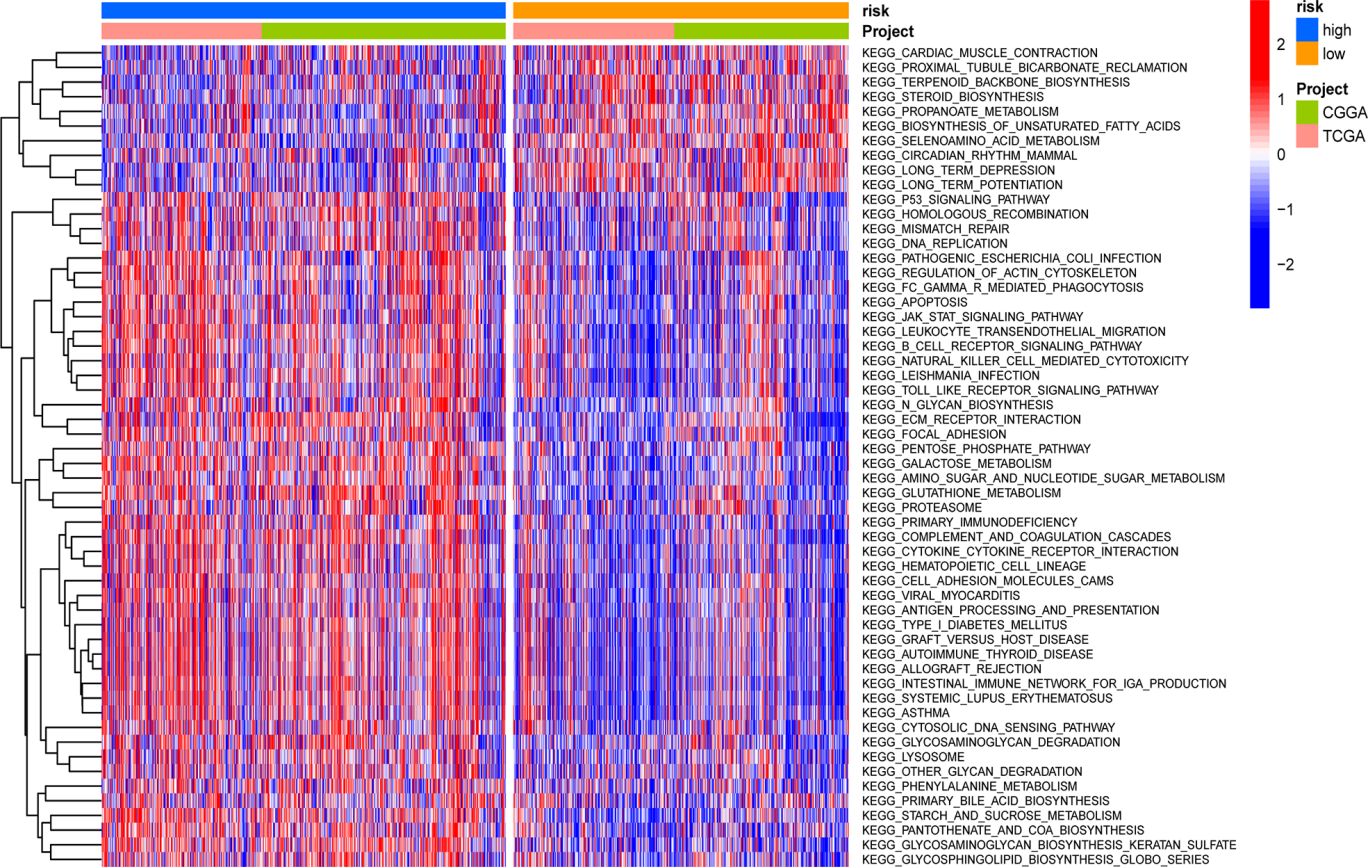
Heatmap for the contribution of gene set variation analysis (GSVA) scores of KEGG in low- and high-risk groups.

GSVA was applied to score differences in pathway activity in different groups. The signaling pathways related to tumorigenesis and oncogenic transformation were mainly enriched in high-risk populations (**Figure 9**), including the P53 pathways and JAK-STAT signaling pathway. These results demonstrate that the GSRS-based risk score, as a new LGG biomarker, may be associated with some important cancer-related signaling pathways.

### Validation of hub genes of GSRS

**Figure 10:**
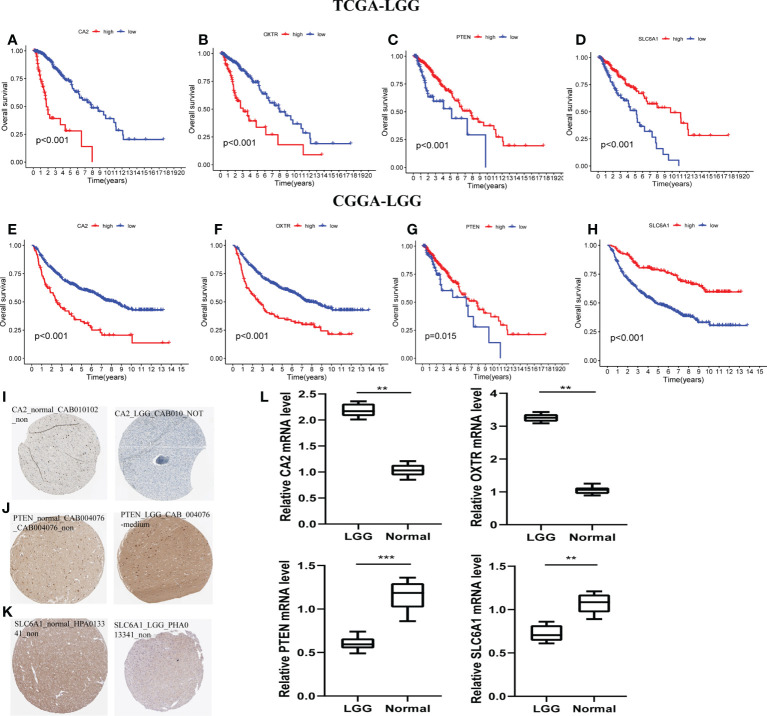
Verification of the prognostic value and expression of hub genes of GSRS. Survival analysis of CA2, OXTR, PTEN, and SLC6A1 for gliomas in TCGA **(A–D)** and CGGA **(E–H)** cohorts. The protein expression level of CA2 **(I)**, PETN **(J)**, and SLC6A1 **(K)** in normal and LGG tissues according to the HPA database. **(L)** The relative mRNA expression levels of CA2, PTEN, SLC6A1, and OXTR are compared among LGG and non-tumor tissues based on real-time PCR results. ***p < 0.001, **p < 0.01.

With the training and validation datasets, we explored the prognostic value of the five-gene GSRS (**Figures 10A** **–H**) (**Table 2**). Expression levels of PTEN and SLC6A1 (**Figures 10C, D**) were positively correlated with the OS in LGGs, but the patients with higher CA2 and OXTR (**Figures 10A, B**) tended to have a worse prognosis based on Kaplan–Meier survival analysis. In addition, CA2, PTEN, SLC6A1, and OXTR were identified as hub genes in the GSRS. Then, the effect of expression of the hub genes on the protein level was evaluated using the HPA database (**Figures 10I** **–K**). The protein PTEN was upregulated and protein SLC6A1 was downregulated in LGGs compared to normal brain tissue, but the expression of protein CA2 was not detected in LGG. Unfortunately, there were no relevant data about OXTR available in the HPA database. In addition, real-time PCR was conducted for the clinical samples at our center (**Figure 10L**). We found that mRNA expressions were upregulated for CA2 and OXTR, but were downregulated for PTEN and SLC6A1 in LGG compared to normal brain tissue. These findings are in accord with those in the public database.

**Table 2:** K–M survival analysis of five GSRS genes in TCGA and CGGA.

Gene		TCGA			CGGA	
	HR (high)		Log-rank *p*	HR (high)		Log-rank *p*
CA2	1.232		*p* < 0.001	1.105		*p* < 0.001
CNTNAP4	0.864		*p* < 0.05	0.893		***p* > 0.05**
SLC6A1	0.683		*p* < 0.001	0.763		*p* < 0.001
PTEN	0.637		*p* < 0.001	0.796		*p* < 0.05
OXTR	1.175		*p* < 0.001	1.268		*p* < 0.001

Factors with p-values more than 0.05 in TCGA and CGGA cohorts are marked in bold.

## Discussion

Gliomas are a type of primary brain tumor with heterogeneous traits. Our understanding of the influence of tumor driver genes on malignant progression has improved as a result of molecular pathology and epidemiological studies, but the association between glioma and the TME remains unclear (29). Preclinical evidence suggests that malignant brain tumor cells could integrate into neural circuits *via* actual brain tumor synapses, and that excitatory neuronal activity would promote brain tumor growth and invasion (30). Moreover, a new study confirmed that specific cell populations in glioblastoma support synaptogenesis to varying degrees (23).

Synapses also play vital roles in many ways associated with the immune system including self-tolerance, adaptive immunity, and prevention of autoimmunity (31). Furthermore, a remarkable feature of solid tumors is the special immune microenvironment, one that might advance the proliferation, invasion, and metastasis of tumor cells. However, it has been suggested that GABAergic synapses might suppress intestinal innate immunity *via* an insulin signal in *Caenorhabditis elegans*, an organism in which a completely unique mechanism by which GABAergic synapses may regulate gut innate immune responses *via* muscle insulin-like signal was discovered (32). Unfortunately, however, there is no specific biomarker constructed primarily according to the GSRGs and immune condition inside gliomas. Our study aimed to construct a five-gene GSRS and evaluate its prognostic role in LGGs.

We also explored the correlation between risk scores and clinicopathological variables and immune profiles. Additionally, the potential molecular mechanism that may be regulated by GSRS was predicted by GSVA. Furthermore, we verified the expression of hub genes in GSRS by real-time PCR of glioma tissues as well as the expression of protein levels in LGG.

We found that the majority of GSRGs were differentially expressed between normal and malignant tissues. Interestingly, the risk score was significantly correlated with the WHO glioma grade, which indicated that it has a high predictive power of malignant degree (33). The five-gene GSRS was developed and verified in our research. A few single-nucleotide polymorphisms (SNPs) and CNVs of GSRGs in LGG indicated that these genes might be associated with glioma progression, and that genome stability was also important in preventing malignant growth. In LGG patients, the risk signature provided a more convenient and exact predictive power than standard clinical prognostic variables. Furthermore, the most common application in clinical practice is the molecular pathologic detection of 1p/19q co-deletion and IDH type, which also could be distinguished with the risk scores. The 1p/19q non-co-deletion and IDH wild-type gliomas predicted a less responsive response to conventional chemoradiotherapy (34). As a result, chemotherapy or radiation may provide less therapeutic benefit for LGGs with higher risk scores.

Tumor-induced dysregulation of immune status may be associated with glioma progression, and the immune components in the immune microenvironment have important functions in glioma progression and prognosis (35, 36). Tumor evolution tends to escape from immune surveillance, especially the tumor-specific immunity that could be affected by the regulation of the immune-related synapse between effector T cells and antigen-presenting cells (37). In our study, M0 macrophages and CD4 memory resting T cells were significantly enriched in LGG. Although gliomas were defined as “cold tumors” with fewer infiltrating immune cells, the proportion of macrophages in the immune microenvironment of gliomas is still as high as 30% to 50% (38). It has been reported that high levels of M2 macrophages (39), neutrophils, and Treg cells in the TME were closely related to poor prognosis in gliomas. Conversely, high levels of M1 macrophages and CD8+ T cells were considered positive factors for gliomas (40). Interestingly, the Tregs and infiltration of M2 macrophages were associated with decreased tumor survival (41). These findings indicated that the patients with high risk were more likely to experience higher M2 and Treg infiltration resulting in poor outcomes. Similarly, the infiltrating level, including immune and stromal scores, was positively associated with the risk score in LGGs. This association demonstrated that a higher fraction of immune-inflammatory tumor-infiltrating cells could establish an immunosuppressive TME in high-risk groups. Hence, to some degree, consuming the number and activity of infiltrating Tregs and the repolarization of M2 into M1 macrophages could be the potential treatments for the LGGs with higher risk scores.

ICPs may inhibit the over-activation of the immune system and prevent the occurrence of allergic reactions and autoimmune diseases (42). In gliomas, common ICPs include PD-1 (43) and PD-L1 (44). Immune checkpoint blockade (ICB) therapy may block the function of checkpoints and reactivate the over-suppressed immune system (45).

It has been reported that ICD could stimulate the immune microenvironment to go from “cold” to “hot” (46). The expression of ICPs is crucial for ICB therapy and immune escape (47). Thus, to some extent, the immune checkpoint inhibitors have become the hotpots of immunotherapy for tumors (48). Hence, targeting immune checkpoint molecules (e.g., CTLA-4, PD-L1, and CD47) that provide inhibitory signals to T cells could significantly improve the survival of patients with refractory tumors. According to our constructed GSRS, the expression of vital ICPs (PD-L1, PD-1, and CTLA4) and TMB was significantly correlated with a risk score, and these indicated that high-risk gliomas were more likely to be sensitive to ICB therapy. For the expression level of ICD, there was no significant difference between the high- and low-risk groups. It is possible that immune infiltration and ICPs could represent a new research direction for predicting the effectiveness of ICB therapy in solid tumors.

When looking for putative mechanisms connected to the GSRS, we also discovered that the highly enriched terms in high-risk samples were primarily cancer-associated pathways. It was found that presynaptic neurons and postsynaptic glioma cells communicate electrochemically through AMPA receptor-dependent synapses (12). It was reported that glutamate may alter glioblastoma malignant progression by stimulating the epidermal growth factor receptor signaling pathway (49). There is relatively little literature detailing the implications of GABAergic signaling in glioma cells in particular. One available report suggests that glioma cells express functional GABA A receptors and that endogenous GABA A receptor activity reduces glioma proliferation ability (19). Apart from the aberrant GABAergic and glutamatergic activity in the glioma microenvironment, the possibilities of inhibiting malignant progression and moderating cognitive damage from radiation treatment by targeting myeloid cells have been reported (50, 51).

In summary, in our study, we constructed a novel prognostic biomarker to predict the role of ICB therapy for LGGs, which could definitely distinguish the immune status and even the malignant degree of glioma. However, the fact cannot be ignored that there are some limitations to applying just a single DEG to predict glioma prognosis, due to the heterogeneous character of this tumor type (49). Similarly, there are still difficulties in distinguishing subtypes of glioma by molecular schedules and classical biotyping methods (52). Additionally, glioma-related electrophysiological research is in the early stages, and more multicenter, prospective, and well-designed trials are greatly needed.

## Conclusions

We constructed and validated a GSRS that included five GSRGs for predicting the prognosis of LGGs. Moreover, by combining immune profiles with genetic multi-omics assays, the GSRS displayed its special abilities for clarifying the mechanisms of the prognosis in LGGs.

## Data availability statement

The original contributions presented in the study are included in the article/**Supplementary Materials**. Further inquiries can be directed to the corresponding authors.

## Ethics statement

Written informed consent was obtained from the individual(s), and minor(s)’ legal guardian/next of kin, for the publication of any potentially identifiable images or data included in this article.

## Author contributions

This manuscript represents original work and has derived from the effort of all the contributing authors. All of them have contributed significantly and are in agreement with the content of the manuscript. QC and FL have contributed to study conception and design. HJ and ZS have contributed to result interpretation and manuscript drafting. HJ has contributed to the IHC experiments and statistical analysis. ZS and FL have contributed to interpretation of the study results and critical revision for important intellectual contents. All authors contributed to the article and approved the submitted version.

## Funding

This work was supported by the National Natural Science Foundation of China (No. 82071299).

## Conflict of interest

The authors declare that the research was conducted in the absence of any commercial or financial relationships that could be construed as a potential conflict of interest.

## Publisher’s note

All claims expressed in this article are solely those of the authors and do not necessarily represent those of their affiliated organizations, or those of the publisher, the editors and the reviewers. Any product that may be evaluated in this article, or claim that may be made by its manufacturer, is not guaranteed or endorsed by the publisher.

## Footnotes

1
http://cancergenome.nih.gov/

2
http://www.cgga.org.cn

3
http://www.broad.mit.edu/gsea/msigdb/

4
http://www.string-db.org/

5
http://www.cbioportal.org/

6
https://www.proteinatlas.org/

## Supplementary material

Supplementary Figure 1 CNV and SNP of GSRGs in LGG according to cBioPortal database. T, tumor; N, normal; CNV, copy number variation; SNP, single-nucleotide polymorphism.

## Abbreviations

SAPs, synapse-associated proteins; CNS, central nervous system; GABAergic, γ-aminobutyric acidergic; LGGs, lower-grade gliomas; TCGA, The Cancer Genome Atlas; CGGA, Chinese Glioma Genome Atlas; GSRGs, γ-aminobutyric acidergic synapse-related genes; GSRS, γ-aminobutyric acidergic synapse-related risk signature; LASSO, least absolute shrinkage and selection operator; TME, tumor microenvironment; ssGSEA, single-sample gene set enrichment analysis; GSVA, gene set variation analysis; HPA, Human Protein Atlas; WHO, World Health Organization; NGS, neuronal glioma synapses; GTEx, Genotype-Tissue Expression; DEGs, differentially expressed genes; CNV, copy number variation; ROC, receiver operating characteristic; ICD, immunogenic cell death; TMB, tumor mutational burden; OS, overall survival; ICPs, immune checkpoints; ICB, immune checkpoint blockade.
