# Supplementary figures and images for: Prognostic value of γ‐aminobutyric acidergic synapse-associated signature for lower-grade gliomas
Source: Front Immunol. 2022 Nov 3;13:983569. doi: 10.3389/fimmu.2022.983569 (PMC9668880; doi:10.3389/fimmu.2022.983569)

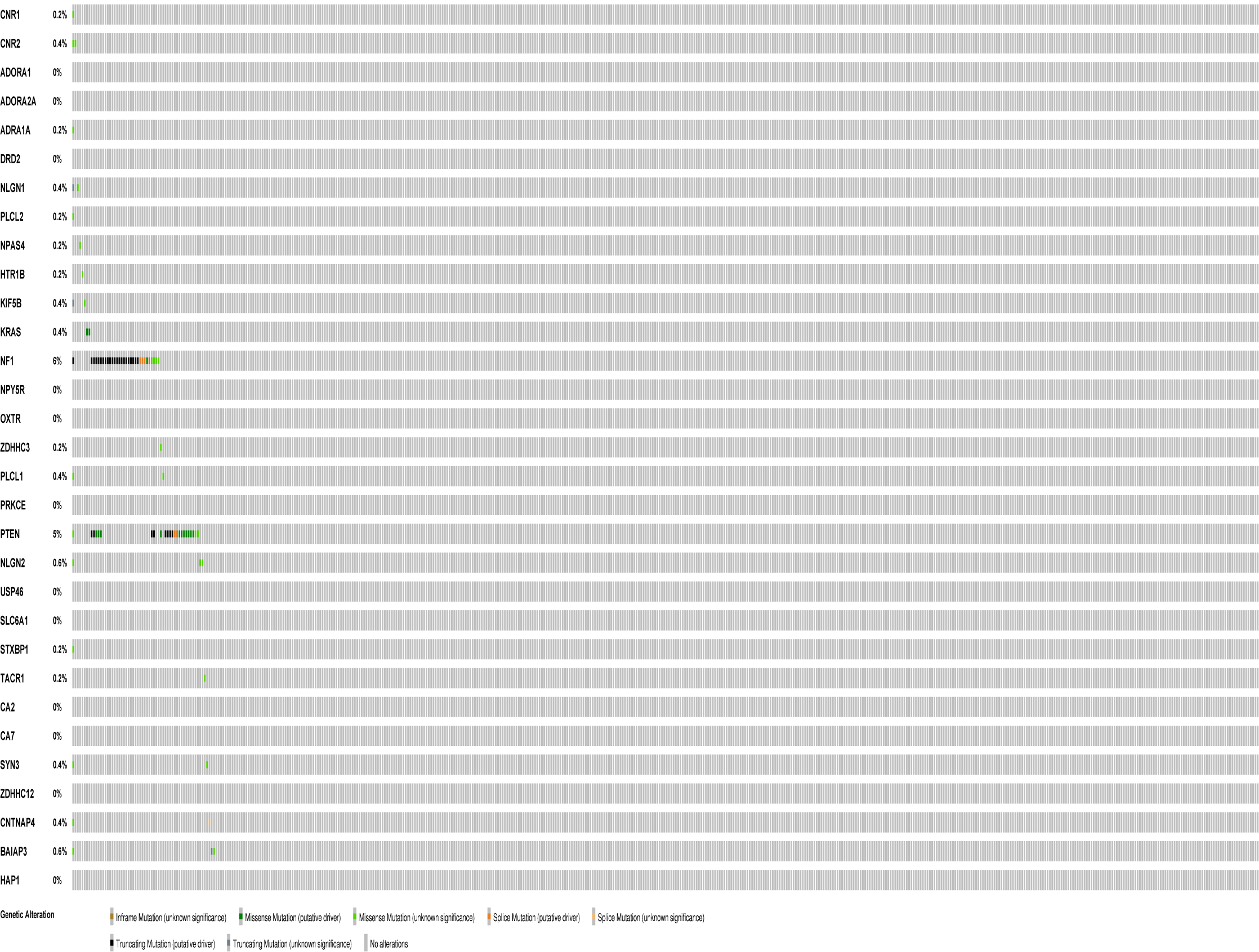

Supplement: Supplementary Figure 1 — CNV and SNP of GSRGs in LGG according to cBioPortal database. T, tumor; N, normal; CNV, copy number variation; SNP, single-nucleotide polymorphism. [file Image_1.tif]
